# Absolute structure of (3a*S*,5*S*,7a*S*,7b*S*,9a*R*,10*R*,12a*R*,12b*S*)-7b-hy­droxy-4,4,7a,9a,12a-penta­methyl-10-[(2′*R*)-6-methyl­heptan-2-yl]-2,8,9-trioxo­octa­deca­hydro­benzo[*d*]indeno­[4,5-*b*]azepin-5-yl acetate from 62-year-old crystals

**DOI:** 10.1107/S205698901901140X

**Published:** 2019-08-23

**Authors:** Leopoldo Suescun, Horacio Heinzen

**Affiliations:** a Cryssmat-Lab/DETEMA, Facultad de Química, Universidad de la República, Av., Gral., Flores 2124, Montevideo 11800, Uruguay; b Departamento de Química Orgánica, Facultad de Química, Universidad de la República, Av. Gral. Flores 2124, Montevideo 11800, Uruguay

**Keywords:** crystal structure, terpenoid, lanosterol, old crystals, disorder

## Abstract

The structure of the title compound, C_32_H_51_NO_6_, was determined from 62-year-old crystals at room temperature and refined with 100 K data in a monoclinic (*C*2) space group. This compound with a triterpenoid structure, now confirmed by this study, played an important role in the determination of the structure of lanosterol. The mol­ecules pack in linear O—H⋯O hydrogen-bonded chains along the short axis (*b*), while parallel chains display weak van der Waals inter­actions that explain the needle-shaped crystal morphology. The structure exhibits disorder of the flexible methyl­heptane chain at one end of the main mol­ecule with a small void around it. Crystals of the compounds were resistant to data collection for decades with the available cameras and Mo *K*α radiation single-crystal diffractometer in our laboratory until a new instrument with Cu *K*α radiation operating at 100 K allowed the structure to be solved and refined.

## Abstract 2

The structure of the title compound was determined using single crystals obtained more than 60 years ago at the Facultad de Química, Universidad de la República. The chemical structure of the compound, now confirmed by X-ray diffraction, was determined spectroscopically and was relevant in the determination of the structure of lanosterol and other triterpenoids in the early 50′s.

## Chemical context   

**Figure 1:**
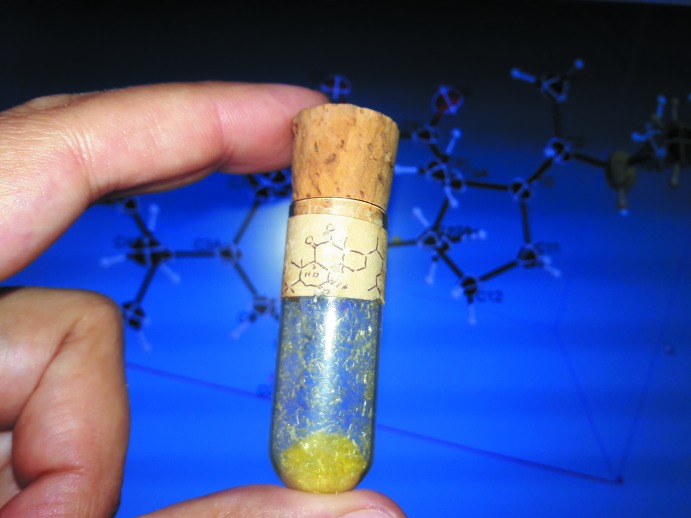
Crystals of the title compound in the original tube where they were saved for more than 60 years.

Crystals of the title compound were obtained by Professor M. R. Falco (1922–2015) in 1952 after a spectroscopic structure determination (Falco *et al.*, 1952 ▸) that was relevant for the correct determination of the structure of lanosterol (Eschenmoser *et al.*, 1955 ▸) and were handed in the glass vial shown in Fig. 1 ▸ to Professor R. Mariezcurrena (1940–2016) in the late 80′s for structure determination by X-ray diffraction. Structure determination was elusive for many years (see the *Supra­molecular features* section for reasons) since the very thin needles available produced no measurable diffraction intensities with the available Weissenberg or Bürger cameras or a sealed-tube Mo *Kα* source diffractometer with a scintillator detector available at the laboratory over that period. The availability of a diffractometer with a Cu *K*α source (acquired and installed at our institution in 2014 during the IYCr) allowed for the determination of the structure at room temperature where significant positional disorder of the terminal aliphatic chain was observed. Data collection at 100 K allowed for the structure refinement reported herein, which confirms the structure determined spectroscopically in the 50′s. Professor Mariezcurrena had the chance to see the final structural model of the RT structure determination before passing away. We dedicate this manuscript to his memory, teachings and patience in keeping the glass vial in a safe place allowing for this report of the successful structure determination.
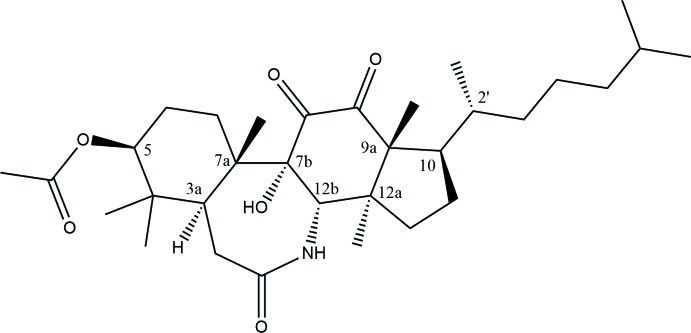


## Structural commentary   

**Figure 2:**
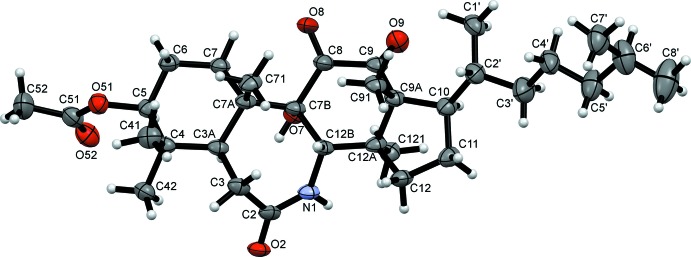
*ORTEP* view of the title compound showing the labelling scheme and displacement ellipsoids drawn at the 50% probability level. The minor occupancy portion of the disordered methyl­heptane mol­ecule is not shown for clarity.

The title compound, shown in Fig. 2 ▸ with the numbering scheme, is a tetra­cyclic triterpenoid with six-, seven-, six and five-membered fused rings, with no insaturations except for three exocyclic carbonyl moieties at C2, C8 and C9. The first two rings define a hydrogenated benzazepine unit while the last two define a hydrogenated indene group. The presence of fused rings of different sizes, one heteroatom and different C-atom hybridization states, together with a large number of exocyclic substituents, leads to a very strained bonding arrangement within the ring system. A full geometrical analysis performed with *Mogul* 1.8.2 (Build 248885) running on the May 2019 update of the CSD (Groom *et al.*, 2016 ▸) shows that all bridgehead atoms in the mol­ecule show atypical bond distances or angles. Table 1 ▸ shows all the bond distances and bond angles that were unexpected according to the *z*-score criterion in *Mogul*. In this table we find that C3*A* and C7*A* (bridgehead atoms in the benzazepine bicycle) C7*B* and C12*B* (bridgehead atoms of the fused azepine and indene groups) and C9*A* (bridgehead atom in the indene bicycle) display unusual bond distances [long C3*A*—C7*A* = 1.584 (4), C7*B*—C8 = 1.580 (4), C7*B*—C12*B* = 1.578 (4) Å and short C9—C9*A* = 1.500 (4) Å] and C7*B*, C9*A* and C12*A* show unusual bond angles [low O7—C7*B*—C8 = 97.7 (2), C12*A*—C9*A*—C9 = 102.5 (2), C12—C12*A*—C9*A =* 101.1 (2)°] in addition to other unusual features.

Another significant contribution to the strain in this region of the mol­ecule is the diketone group C7*B*—C8(=O8)—C9(=O9)—C9*A* that also shows an elongated C*sp^2^*—C*sp^2^* bond [C8—C9 of 1.549 (5) Å] and a large O8—C8—C9—O9 torsion angle of −43.7 (5)°. Repulsion between O8 and O9 leads to the increase of the torsion angle in the *cis* diketone group and lengthening of the C8—C9 bond distance, contributing to the unusual conformation of the C7*B*/C8/C9/C9*A*/C12*A*/C12*B* ring. Puckering parameters for this ring are θ = 149°(or 31° considering the inverted order of atoms) and Φ = 13.5°, which fall far from all usual parameters for frequently observed geometries of six-membered rings, between a chair and a half-chair conformation, confirming the effects of the observed bond distances and angles. Considering a distorted chair conformation, atom C7*B* is only 0.394 (5) Å away from the C8/C9/C12*A*/C12*B* plane [maximum deviation of 0.0382 (17) Å], while C9*A* is on the other side of the plane, displaced by 0.821 (4) Å. The seven-membered ring shows a chair conformation with atoms N1 and C1 lying 1.163 (5) and 1.137 (4) Å, respectively, above and C7*A* 0.721 (4) Å below the almost planar C3/C3*A*/C12*B*/C7*B* group of atoms [maximum deviation of 0.038 (1) Å for C3*A*]. The five-membered ring exhibits a half-chair conformation with puckering parameter Φ = 344.2 (5)°, atom C12*A* lying 0.655 (5) Å away from the C9*A/*C10–C12 plane [maximum deviation of 0.094 (2) Å for C11]. The remaining six-membered ring (C3*A*/C4–C7/C7*A*) has puckering parameters θ = 170° and Φ = 326° with atoms C5 and C7*A* located 0.703 (4) and −0.617 (5) Å, respectively, away from the C3*A*/C4/C6/C7 plane [maximum deviation of 0.0271 (16) Å for C6]. The conformation of the four rings, with most of the substituents in an equatorial configuration, makes the ring system almost planar with maximum deviations for N1 and C3 [0.794 (3) and −0.616 (3) Å, respectively] on either side. The six-methyl heptane chain C1′ to C8′ shows positional disorder, modelled over two sites with occupancies of 0.819 (6) and 0.181 (6), around a structural void of 36 Å^3^ surrounded by equivalent aliphatic chains. At room temperature, this chain could not be modelled properly over two sites.

## Supra­molecular features   

**Figure 3:**
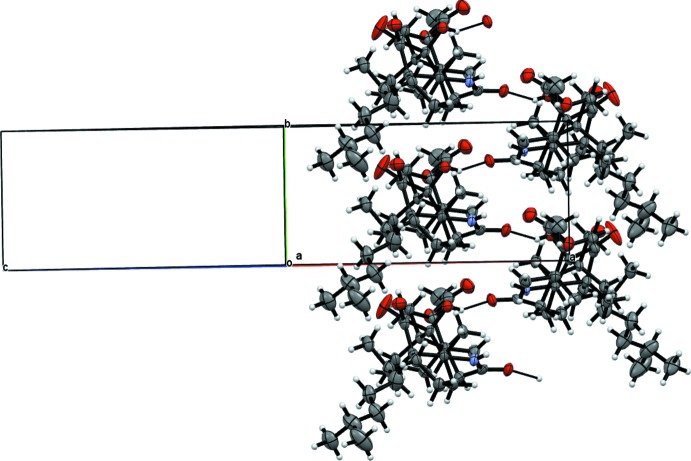
Zigzag chains of mol­ecules connected by O7—H7⋯O2(−*x* + 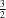, *y* + 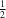, −*z*) inter­actions along the *b-*axis direction.

**Figure 4:**
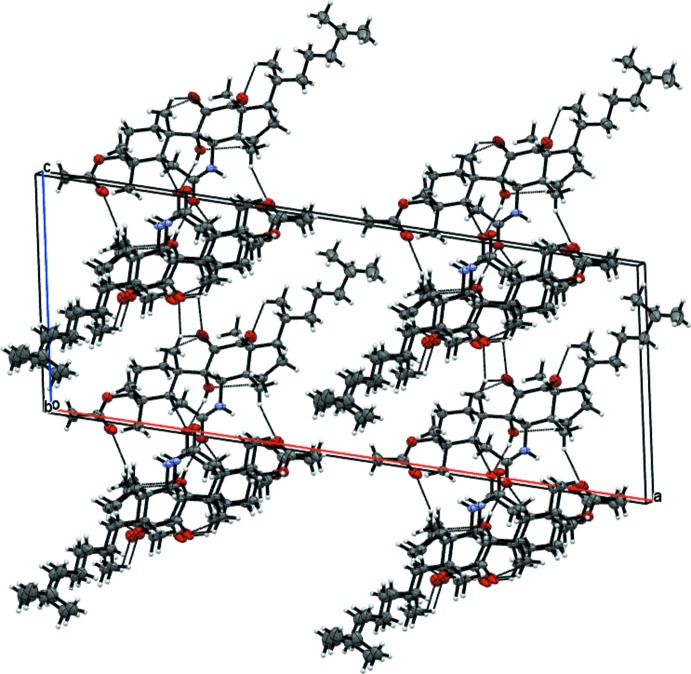
View of the packing of the title compound along [010] showing the formation of C—H⋯O hydrogen-bonded planes and the voids left by symmetry-related methyl­heptane chains.

In the crystal, mol­ecules of the title compound pack in an elongated conformation laying parallel to the [102] direction. The packing is directed by O7—H7⋯O2^i^ hydrogen bonds (see Table 2 ▸, Fig. 3 ▸), forming zigzag chains running along the [010] direction (determined using *PLATON* software; Spek, 2009 ▸). These chains are connected in the [001] direction through weak C—H⋯O inter­actions: C71—H71*A*⋯O8^ii^ and C121—H12*E*⋯O52^iii^ with H71*A*⋯O8^ii^ and H12*E*⋯O52^iii^ distances of 2.55 and 2.53 Å, respectively [symmetry codes: (ii) −*x* + 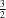, *y* − 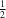, −*z* + 1; (iii) −*x* + 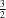, *y* − 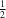, −*z*]. These inter­actions define double planes of mol­ecules with the polar regions of the mol­ecules in contact, leaving the terminal aliphatic chains pointing outwards. Parallel planes are only weakly bound by dispersion forces: indeed, voids of *ca* 39 Å^3^ are found between non-polar residues from parallel planes (Fig. 4 ▸). These strong inter­actions along [010], weak along [001] and very weak along [100] nicely explain the flat needle crystal shape observed where face indexing suggests the needle length and longer dimension of the largest planes is [010], the shorter dimension of the planes is [001] and the very narrow width of the crystals is [100]. The large unit cell, the presence of positional disorder (aggravated at room temperature) and voids in the crystal structure, combined with the C, H, N and O composition of the crystals explain the poor scattering power that prevented structure determination with older instruments.

## Database survey   

The May 2019 update of the CSD (Groom *et al.*, 2016 ▸) contains no compounds displaying the same arrangement of six-, seven-, six- and five-membered rings (disregarding bond type) with an N atom in the seven-membered ring. Three penta­cyclic compounds with a tetra­zole ring at N1—C2 have been reported [LEXVOB (Alam *et al.*, 2013 ▸), TZANDT (Husain *et al.*, 1981 ▸) and VEVLAK (Rajnikant *et al.*, 2006 ▸)], the former and latter showing very similar mol­ecular conformation and inter­actions that lead to very similar unit-cell dimensions (∼35×6×12 Å). There is only one entry with the same six-, seven-, six- and five-membered ring combination containing O instead of N (HIXSAI; Morales *et al.*, 1999 ▸) but the configuration of C5 is inverted and therefore the dihedral angle between mean planes of the six- and seven-membered rings differ significantly and thus also the mol­ecular conformation. There are also three tetra­cyclic compounds with no heteroatom in the ring arrangement [OQIVIU (Kranz *et al.*, 2011 ▸), UBEDIO (Wang *et al.*, 2000 ▸) and WECQAY (Kranz *et al.*, 2012 ▸)] all showing very different stereochemistry; therefore, the mol­ecular conformations are not comparable. This is, therefore, the first report of this 6-7-6-5 ring system containing the azepine ring.

## Synthesis and crystallization   

Synthesis and crystallization were reported by Falco *et al.* (1952 ▸). Crystals were not recrystallized after the initial preparation.

## Refinement   

Crystal data, data collection and structure refinement details are summarized in Table 3 ▸. C- and N-bound H atoms were placed in calculated positions (C—H = 0.93–0.99, N—H = 0.87 Å) and included as riding contributions. The OH H atom was found in a difference-Fourier map and refined as riding with a rotating torsion angle and O—H distance restraint. All H atoms were refined with isotropic displacement parameters set at 1.2–1.5 times the *U_eq_* value of the parent atom.

## Supplementary Material

Crystal structure: contains datablock(s) I. DOI: 10.1107/S205698901901140X/ex2022sup1.cif

Structure factors: contains datablock(s) I. DOI: 10.1107/S205698901901140X/ex2022Isup2.hkl

CCDC reference: 1946967

Additional supporting information:  crystallographic information; 3D view; checkCIF report

## Figures and Tables

**Table 1:** Unusual bond distances and bond angles (φ) (Å,°) extracted from *Mogul* *z*-score = |*d* − *d*_mean_|/SD, where *d*_mean_ and SD are the mean and standard deviation of *N* observed values in the *Mogul* database. The *z*-score for bond angles is calculated replacing *d* by φ. A bond distance or angle is considered unusual if the *z*-score > 2.

Bond	*N*	bond distance (*d*)	*d*_mean_	SD	*z*-score
C7*A*—C3*A*	563	1.584	1.559	0.011	2.211
C7*B*—C8	16	1.579	1.529	0.008	6.313
C9*A*—C9	20	1.500	1.520	0.009	2.374
C7*B*—C12*B*	18	1.579	1.547	0.016	1.957^*a*^
					
Angle	*N*	φ	φ_mean_	SD	*z*-score
C3*A*—C3—C2	5	107.1	114.1	3.6	2.088
O51—C5—C4	158	110.2	107.8	1.2	2.067
O7—C7*B*—C8	16	97.7	105.7	3.7	2.131
O8—C8—C9	18	115.9	120.9	1.3	3.773
C9*A*—C9—C8	28	112.3	118.3	1.8	2.577
O9—C9—C9*A*	20	128.7	122.9	2.2	2.138
C12*A*—C9*A*—C9	15	102.5	110.0	3.2	2.369
C12—C11—C10	894	108.0	104.4	1.7	2.138
C12—C12*A*—C9*A*	13	101.14	103.11	0.87	2.264
C121—C12*A*—C9*A*	8	110.1	113.2	1.4	2.136

Note: (*a*) This value is lower than 2 but this bond is still unusually long and relevant for the discussion.

**Table 2:** Hydrogen-bond geometry (Å, °)

*D*—H⋯*A*	*D*—H	H⋯*A*	*D*⋯*A*	*D*—H⋯*A*
O7—H7⋯O2^i^	0.79 (5)	2.02 (5)	2.750 (3)	153 (5)
C3*A*—H3*C*⋯O2^i^	1.00	2.18	3.173 (4)	176
C121—H12*E*⋯O52^ii^	0.98	2.53	3.428 (4)	153
C71—H71*A*⋯O8^iii^	0.98	2.55	3.224 (4)	126
C91—H91*B*⋯O9^iv^	0.98	2.48	3.422 (5)	162

Symmetry codes: (i) 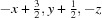; (ii) 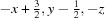; (iii) 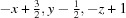; (iv) 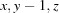.

**Table 3:** Experimental details

Crystal data	
Chemical formula	C_32_H_51_NO_6_
*M*_r_	545.73
Crystal system, space group	Monoclinic, *C*2
Temperature (K)	100
*a*, *b*, *c* (Å)	34.882 (4), 6.5332 (11), 13.6021 (16)
β (°)	100.599 (14)
*V* (Å^3^)	3046.9 (7)
*Z*	4
Radiation type	Cu *K*α
μ (mm^−1^)	0.64
Crystal size (mm)	0.44 × 0.33 × 0.09

Data collection	
Diffractometer	Bruker D8 Venture
Absorption correction	Multi-scan (*SADABS*; Krause *et al.*, 2015 ▸)
*T*_min_, *T*_max_	0.804, 0.946
No. of measured, independent and observed [*I* > 2σ(*I*)] reflections	14487, 5509, 5056
*R*_int_	0.034
(sin θ/λ)_max_ (Å^−1^)	0.603

Refinement	
*R*[*F* ^2^ > 2σ(*F* ^2^)], *wR*(*F* ^2^), *S*	0.050, 0.135, 1.07
No. of reflections	5509
No. of parameters	410
No. of restraints	179
H-atom treatment	H atoms treated by a mixture of independent and constrained refinement
Δρ_max_, Δρ_min_ (e Å^−3^)	0.41, −0.20
Absolute structure	Flack *x* determined using 2070 quotients [(*I* ^+^)−(*I* ^−^)]/[(*I* ^+^)+(*I* ^−^)] (Parsons et al., 2013 ▸)
Absolute structure parameter	0.09 (10)

Computer programs: *APEX2* and *SAINT* (Bruker, 2014 ▸), *SHELXT* (Sheldrick, 2015 ▸a), *SHELXL2018* (Sheldrick, 2015 ▸b), *Mercury* (Macrae *et al.*, 2008 ▸) and *publCIF* (Westrip, 2010 ▸).

## supplementary crystallographic information

### Crystal data

**Table:**

C_32_H_51_NO_6_	*F*(000) = 1192
*M**_r_* = 545.73	*D*_x_ = 1.190 Mg m^−^^3^
Monoclinic, *C*2	Cu *K*α radiation, λ = 1.54178 Å
*a* = 34.882 (4) Å	Cell parameters from 95 reflections
*b* = 6.5332 (11) Å	θ = 12.3–47.3°
*c* = 13.6021 (16) Å	µ = 0.64 mm^−^^1^
β = 100.599 (14)°	*T* = 100 K
*V* = 3046.9 (7) Å^3^	Flat needles, yellow
*Z* = 4	0.44 × 0.33 × 0.09 mm

### Data collection

**Table:**

Bruker D8 Venture diffractometer	5509 independent reflections
Radiation source: Incoatec I microsource	5056 reflections with *I* > 2σ(*I*)
Detector resolution: 10.4167 pixels mm^-1^	*R*_int_ = 0.034
ω and φ scans	θ_max_ = 68.4°, θ_min_ = 2.6°
Absorption correction: multi-scan (SADABS; Krause *et al.*, 2015)	*h* = −38→42
*T*_min_ = 0.804, *T*_max_ = 0.946	*k* = −7→7
14487 measured reflections	*l* = −14→16

### Refinement

**Table:**

Refinement on *F*^2^	Secondary atom site location: difference Fourier map
Least-squares matrix: full	Hydrogen site location: mixed
*R*[*F*^2^ > 2σ(*F*^2^)] = 0.050	H atoms treated by a mixture of independent and constrained refinement
*wR*(*F*^2^) = 0.135	*w* = 1/[σ^2^(*F*_o_^2^) + (0.0771*P*)^2^ + 1.6616*P*] where *P* = (*F*_o_^2^ + 2*F*_c_^2^)/3
*S* = 1.07	(Δ/σ)_max_ = 0.001
5509 reflections	Δρ_max_ = 0.41 e Å^−^^3^
410 parameters	Δρ_min_ = −0.19 e Å^−^^3^
179 restraints	Absolute structure: Flack *x* determined using 2070 quotients [(*I*^+^)-(*I*^-^)]/[(*I*^+^)+(*I*^-^)] (Parsons *et al.*, 2013)
Primary atom site location: dual	Absolute structure parameter: 0.09 (10)

### Special details

**Table:**

Geometry. Least-squares planes (x,y,z in crystal coordinates) and deviations from them (* indicates atom used to define plane)14.9003(0.0756)x + 4.8675(0.0079)y - 7.9183(0.0216)z = 11.1840(0.0683) * -0.0587 (0.0014) C9A * 0.0916 (0.0021) C10 * -0.0937 (0.0022) C11 * 0.0609 (0.0015) C12 0.6554 (0.0051) C12A Rms deviation of fitted atoms = 0.0780 10.0303(0.0705)x + 4.8288(0.0060)y - 8.8634(0.0137)z = 7.4252(0.0575) Angle to previous plane (with approximate esd) = 9.752 ( 0.251 ) * 0.0382 (0.0017) C12A * -0.0354 (0.0015) C12B * 0.0351 (0.0015) C8 * -0.0379 (0.0017) C9 -0.8207 (0.0043) C9A 0.3944 (0.0051) C7B Rms deviation of fitted atoms = 0.0367 14.5476(0.0446)x + 3.1069(0.0108)y - 11.3991(0.0111)z = 9.7791(0.0311) Angle to previous plane (with approximate esd) = 19.404 ( 0.209 ) * -0.0351 (0.0015) C3 * 0.0382 (0.0016) C3A * -0.0374 (0.0016) C7B * 0.0343 (0.0015) C12B -0.7211 (0.0042) C7A 1.1626 (0.0045) C2 1.1371 (0.0040) N1 Rms deviation of fitted atoms = 0.03636.8164(0.0691)x + 4.5554(0.0061)y - 9.7096(0.0126)z = 5.1714(0.0475) Angle to previous plane (with approximate esd) = 18.692 ( 0.202 ) * -0.0271 (0.0016) C6 * 0.0261 (0.0015) C4 * 0.0270 (0.0016) C7 * -0.0261 (0.0015) C3A 0.7034 (0.0044) C5 -0.6172 (0.0045) C7A Rms deviation of fitted atoms = 0.026610.0303(0.0705)x + 4.8288(0.0060)y - 8.8634(0.0137)z = 7.4252(0.0575) Angle to previous plane (with approximate esd) = 7.415 ( 0.200 ) * 0.0382 (0.0017) C12A * -0.0354 (0.0015) C12B * 0.0351 (0.0015) C8 * -0.0379 (0.0017) C9 -0.8207 (0.0043) C9A 0.3944 (0.0051) C7B 0.1064 (0.0121) C4 0.4530 (0.0094) C7 0.0351 (0.0015) C8 0.0382 (0.0017) C12A Rms deviation of fitted atoms = 0.0367 14.4178(0.0111)x + 4.8025(0.0030)y - 8.2192(0.0070)z = 10.9550(0.0080) Angle to previous plane (with approximate esd) = 8.306 ( 0.172 ) * -0.3830 (0.0029) C9A * -0.2601 (0.0031) C10 * -0.4143 (0.0034) C11 * -0.2168 (0.0034) C12 * 0.3709 (0.0030) C12A * 0.0762 (0.0030) C12B * 0.4094 (0.0031) C7B * 0.1967 (0.0033) C8 * 0.3447 (0.0029) C9 * -0.1036 (0.0028) C3A * -0.6156 (0.0029) C3 * 0.5099 (0.0029) C2 * 0.7943 (0.0028) N1 * -0.3837 (0.0029) C7A * 0.1748 (0.0031) C7 * -0.2604 (0.0029) C6 * 0.2105 (0.0029) C5 * -0.4499 (0.0028) C4 Rms deviation of fitted atoms = 0.3855 All esds (except the esd in the dihedral angle between two l.s. planes) are estimated using the full covariance matrix. The cell esds are taken into account individually in the estimation of esds in distances, angles and torsion angles; correlations between esds in cell parameters are only used when they are defined by crystal symmetry. An approximate (isotropic) treatment of cell esds is used for estimating esds involving l.s. planes.

### Fractional atomic coordinates and isotropic or equivalent isotropic displacement parameters (Å^2^)

**Table:**

	*x*	*y*	*z*	*U*_iso_*/*U*_eq_	Occ. (<1)
N1	0.79213 (8)	0.2991 (4)	0.13482 (19)	0.0333 (6)	
H1	0.8127 (12)	0.336 (7)	0.112 (3)	0.040*	
C2	0.76039 (9)	0.2296 (5)	0.0731 (2)	0.0328 (7)	
O2	0.75912 (7)	0.2260 (4)	−0.01801 (15)	0.0388 (5)	
C3	0.72638 (10)	0.1796 (5)	0.1211 (2)	0.0352 (7)	
H3A	0.734863	0.096037	0.181978	0.042*	
H3B	0.706632	0.101249	0.074346	0.042*	
C3A	0.70900 (9)	0.3854 (5)	0.1486 (2)	0.0306 (7)	
H3C	0.718787	0.487501	0.104353	0.037*	
C4	0.66354 (10)	0.3882 (5)	0.1126 (2)	0.0336 (7)	
C41	0.64140 (10)	0.2211 (6)	0.1579 (3)	0.0443 (8)	
H41A	0.654842	0.089874	0.155829	0.066*	
H41B	0.614821	0.210457	0.119331	0.066*	
H41C	0.640359	0.255936	0.227404	0.066*	
C42	0.65520 (10)	0.3588 (6)	−0.0024 (2)	0.0394 (8)	
H42A	0.659808	0.215645	−0.018299	0.059*	
H42B	0.672523	0.447548	−0.032513	0.059*	
H42C	0.627994	0.394661	−0.029059	0.059*	
C5	0.65000 (9)	0.6039 (5)	0.1346 (2)	0.0330 (7)	
H5	0.662480	0.704375	0.094807	0.040*	
O51	0.60807 (7)	0.6204 (4)	0.10484 (17)	0.0401 (5)	
C51	0.59429 (11)	0.7431 (6)	0.0266 (3)	0.0442 (8)	
O52	0.61444 (8)	0.8312 (5)	−0.0233 (2)	0.0529 (7)	
C52	0.55092 (12)	0.7562 (8)	0.0117 (4)	0.0652 (12)	
H52A	0.541075	0.833687	−0.049392	0.098*	
H52B	0.543343	0.825485	0.069126	0.098*	
H52C	0.539831	0.617917	0.005627	0.098*	
C6	0.66096 (9)	0.6595 (5)	0.2436 (2)	0.0353 (7)	
H6A	0.650464	0.555997	0.284763	0.042*	
H6B	0.649609	0.794079	0.255533	0.042*	
C7	0.70501 (9)	0.6684 (5)	0.2732 (2)	0.0343 (7)	
H7A	0.711885	0.708535	0.344415	0.041*	
H7B	0.714916	0.776287	0.233210	0.041*	
C7A	0.72588 (10)	0.4639 (5)	0.2582 (2)	0.0306 (7)	
C71	0.71918 (11)	0.3108 (6)	0.3390 (2)	0.0408 (8)	
H71A	0.730916	0.363841	0.405125	0.061*	
H71B	0.731235	0.179355	0.327880	0.061*	
H71C	0.691110	0.291401	0.335717	0.061*	
C7B	0.77147 (10)	0.5111 (5)	0.2693 (2)	0.0317 (7)	
O7	0.77907 (6)	0.6857 (3)	0.21275 (17)	0.0335 (5)	
H7	0.7707 (12)	0.658 (7)	0.156 (4)	0.050*	
C8	0.78966 (10)	0.5958 (6)	0.3765 (2)	0.0391 (8)	
O8	0.77191 (8)	0.6927 (7)	0.4278 (2)	0.0829 (13)	
C9	0.83368 (10)	0.5661 (5)	0.4184 (2)	0.0335 (7)	
C9A	0.84696 (9)	0.3514 (5)	0.4047 (2)	0.0317 (7)	
C91	0.82138 (9)	0.2065 (6)	0.4547 (2)	0.0360 (7)	
H91A	0.826979	0.227632	0.527272	0.054*	
H91B	0.827107	0.064266	0.439666	0.054*	
H91C	0.793800	0.235550	0.428959	0.054*	
C10	0.89081 (10)	0.2995 (6)	0.4364 (2)	0.0379 (7)	
H10	0.906192	0.420551	0.420816	0.045*	
C11	0.89624 (11)	0.1245 (6)	0.3625 (3)	0.0434 (8)	
H11A	0.920446	0.146736	0.335681	0.052*	
H11B	0.898237	−0.009059	0.397388	0.052*	
C12	0.86066 (11)	0.1257 (6)	0.2767 (2)	0.0406 (8)	
H12F	0.868963	0.118140	0.211007	0.049*	
H12G	0.843224	0.008637	0.282793	0.049*	
C12A	0.84002 (9)	0.3296 (5)	0.2881 (2)	0.0322 (7)	
C12B	0.79618 (9)	0.3230 (5)	0.2432 (2)	0.0302 (6)	
H12B	0.785194	0.197165	0.269439	0.036*	
O9	0.85249 (7)	0.7097 (4)	0.4556 (2)	0.0465 (6)	
C121	0.86154 (10)	0.5033 (6)	0.2422 (2)	0.0384 (8)	
H12C	0.888954	0.506642	0.275413	0.058*	
H12D	0.849249	0.634979	0.251622	0.058*	
H12E	0.859974	0.477879	0.170555	0.058*	
C1'	0.90211 (11)	0.4248 (7)	0.6163 (3)	0.0466 (9)	
H1A'	0.912000	0.385993	0.685909	0.070*	
H1B'	0.874761	0.466870	0.608998	0.070*	
H1C'	0.917589	0.538806	0.597679	0.070*	
C2'	0.90518 (10)	0.2420 (7)	0.5480 (3)	0.0452 (8)	
H2'	0.887713	0.131652	0.565422	0.054*	
C3'	0.94661 (13)	0.1581 (9)	0.5657 (3)	0.0667 (12)	0.819 (6)
H3A'	0.964966	0.270218	0.558402	0.080*	0.819 (6)
H3B'	0.948759	0.053055	0.514555	0.080*	0.819 (6)
C4'	0.95816 (14)	0.0619 (10)	0.6714 (4)	0.0536 (13)	0.819 (6)
H4A'	0.963303	0.172770	0.721721	0.064*	0.819 (6)
H4B'	0.936054	−0.020350	0.686075	0.064*	0.819 (6)
C5'	0.99339 (15)	−0.0711 (10)	0.6805 (5)	0.0642 (15)	0.819 (6)
H5A'	1.013473	0.003536	0.651906	0.077*	0.819 (6)
H5B'	0.986294	−0.194616	0.638929	0.077*	0.819 (6)
C6'	1.01156 (19)	−0.1401 (11)	0.7854 (5)	0.0712 (19)	0.819 (6)
H6'	1.018694	−0.014328	0.826567	0.085*	0.819 (6)
C7'	0.98079 (16)	−0.2634 (12)	0.8343 (5)	0.0672 (17)	0.819 (6)
H7A'	0.992903	−0.306986	0.901833	0.101*	0.819 (6)
H7B'	0.972100	−0.384019	0.793412	0.101*	0.819 (6)
H7C'	0.958335	−0.175495	0.837979	0.101*	0.819 (6)
C8'	1.04726 (19)	−0.2635 (16)	0.7908 (7)	0.106 (3)	0.819 (6)
H8A'	1.057945	−0.295880	0.860823	0.159*	0.819 (6)
H8B'	1.066568	−0.185930	0.762027	0.159*	0.819 (6)
H8C'	1.041015	−0.390809	0.753127	0.159*	0.819 (6)
C3M'	0.94661 (13)	0.1581 (9)	0.5657 (3)	0.0667 (12)	0.181 (6)
H3C'	0.959002	0.233500	0.516717	0.080*	0.181 (6)
H3D'	0.943250	0.016366	0.539797	0.080*	0.181 (6)
C4M'	0.9799 (5)	0.138 (3)	0.6583 (12)	0.059 (3)	0.181 (6)
H4C'	1.005556	0.123911	0.637681	0.070*	0.181 (6)
H4D'	0.980638	0.259273	0.702512	0.070*	0.181 (6)
C5M'	0.9698 (6)	−0.051 (4)	0.7099 (17)	0.068 (4)	0.181 (6)
H5C'	0.963486	−0.159040	0.658307	0.081*	0.181 (6)
H6D'	0.945500	−0.022405	0.735326	0.081*	0.181 (6)
C6M'	0.9986 (6)	−0.141 (3)	0.7951 (14)	0.071 (4)	0.181 (6)
H6M'	0.992468	−0.084589	0.858787	0.085*	0.181 (6)
C7M'	0.9925 (9)	−0.382 (3)	0.797 (3)	0.102 (8)	0.181 (6)
H7D'	1.006914	−0.437939	0.859637	0.153*	0.181 (6)
H7E'	1.002258	−0.444041	0.740411	0.153*	0.181 (6)
H7F'	0.964712	−0.412719	0.790954	0.153*	0.181 (6)
C8M'	1.0400 (6)	−0.100 (6)	0.794 (3)	0.104 (8)	0.181 (6)
H8D'	1.056040	−0.154976	0.855335	0.156*	0.181 (6)
H8E'	1.044173	0.048171	0.791197	0.156*	0.181 (6)
H8F'	1.047478	−0.165404	0.735748	0.156*	0.181 (6)

### Atomic displacement parameters (Å^2^)

**Table:**

	*U*^11^	*U*^22^	*U*^33^	*U*^12^	*U*^13^	*U*^23^
N1	0.0447 (15)	0.0362 (14)	0.0207 (13)	0.0050 (12)	0.0102 (11)	−0.0015 (11)
C2	0.0479 (17)	0.0257 (14)	0.0255 (15)	0.0077 (14)	0.0082 (13)	−0.0045 (12)
O2	0.0531 (13)	0.0422 (13)	0.0216 (11)	0.0068 (11)	0.0078 (9)	−0.0066 (10)
C3	0.0479 (18)	0.0279 (15)	0.0296 (16)	0.0002 (14)	0.0064 (13)	−0.0020 (12)
C3A	0.0426 (17)	0.0285 (15)	0.0215 (14)	0.0009 (13)	0.0081 (12)	0.0014 (12)
C4	0.0414 (18)	0.0324 (16)	0.0284 (16)	−0.0033 (14)	0.0102 (13)	−0.0009 (13)
C41	0.0492 (19)	0.0392 (18)	0.046 (2)	−0.0052 (17)	0.0134 (15)	0.0025 (16)
C42	0.0433 (18)	0.0434 (19)	0.0303 (17)	−0.0023 (15)	0.0034 (13)	−0.0047 (14)
C5	0.0349 (16)	0.0364 (16)	0.0279 (16)	−0.0022 (14)	0.0061 (12)	0.0011 (13)
O51	0.0365 (12)	0.0477 (13)	0.0359 (12)	0.0013 (10)	0.0060 (9)	0.0032 (10)
C51	0.0500 (19)	0.0427 (19)	0.0364 (18)	0.0023 (16)	−0.0012 (15)	−0.0024 (15)
O52	0.0624 (16)	0.0534 (16)	0.0389 (14)	0.0012 (14)	−0.0009 (12)	0.0102 (12)
C52	0.046 (2)	0.078 (3)	0.066 (3)	0.014 (2)	−0.0057 (19)	−0.007 (2)
C6	0.0419 (17)	0.0376 (17)	0.0276 (16)	0.0061 (14)	0.0101 (12)	0.0000 (13)
C7	0.0425 (17)	0.0375 (17)	0.0230 (14)	0.0052 (14)	0.0066 (12)	−0.0034 (13)
C7A	0.0422 (17)	0.0322 (15)	0.0185 (14)	0.0041 (13)	0.0080 (12)	0.0012 (12)
C71	0.0511 (19)	0.0480 (19)	0.0263 (16)	0.0096 (17)	0.0146 (14)	0.0078 (15)
C7B	0.0453 (18)	0.0321 (16)	0.0172 (14)	0.0064 (14)	0.0048 (12)	−0.0016 (12)
O7	0.0410 (12)	0.0309 (11)	0.0268 (11)	0.0015 (9)	0.0013 (9)	−0.0003 (9)
C8	0.0478 (19)	0.0443 (18)	0.0240 (16)	0.0122 (16)	0.0033 (13)	−0.0064 (14)
O8	0.0574 (16)	0.139 (3)	0.0445 (16)	0.042 (2)	−0.0106 (13)	−0.051 (2)
C9	0.0451 (19)	0.0354 (17)	0.0208 (14)	0.0023 (14)	0.0080 (13)	0.0001 (12)
C9A	0.0411 (17)	0.0335 (16)	0.0209 (14)	0.0053 (13)	0.0068 (12)	0.0037 (12)
C91	0.0460 (17)	0.0408 (17)	0.0214 (14)	0.0025 (16)	0.0064 (12)	0.0056 (13)
C10	0.0421 (18)	0.0443 (18)	0.0280 (17)	0.0088 (15)	0.0083 (13)	0.0058 (14)
C11	0.049 (2)	0.0478 (19)	0.0349 (18)	0.0173 (17)	0.0106 (14)	0.0058 (16)
C12	0.055 (2)	0.0412 (18)	0.0270 (16)	0.0173 (16)	0.0111 (14)	0.0002 (14)
C12A	0.0438 (17)	0.0326 (16)	0.0221 (15)	0.0077 (14)	0.0109 (12)	0.0027 (12)
C12B	0.0427 (17)	0.0307 (15)	0.0181 (14)	0.0040 (14)	0.0077 (12)	0.0010 (12)
O9	0.0492 (14)	0.0379 (13)	0.0503 (14)	0.0015 (12)	0.0036 (11)	−0.0011 (12)
C121	0.0401 (18)	0.0472 (19)	0.0297 (16)	0.0076 (15)	0.0111 (13)	0.0100 (15)
C1'	0.046 (2)	0.061 (2)	0.0303 (18)	−0.0031 (18)	0.0013 (14)	0.0018 (16)
C2'	0.0460 (18)	0.057 (2)	0.0310 (17)	0.0100 (17)	0.0036 (14)	0.0074 (16)
C3'	0.058 (2)	0.092 (3)	0.047 (2)	0.025 (2)	−0.0009 (17)	0.008 (2)
C4'	0.040 (2)	0.081 (3)	0.039 (2)	0.002 (2)	0.0031 (19)	0.007 (2)
C5'	0.041 (3)	0.085 (4)	0.069 (3)	0.005 (3)	0.014 (2)	0.026 (3)
C6'	0.056 (4)	0.076 (4)	0.075 (4)	−0.013 (3)	−0.005 (3)	0.026 (3)
C7'	0.053 (3)	0.087 (4)	0.058 (3)	0.015 (3)	0.000 (2)	0.023 (3)
C8'	0.064 (4)	0.127 (7)	0.127 (6)	0.017 (4)	0.019 (4)	0.072 (6)
C3M'	0.058 (2)	0.092 (3)	0.047 (2)	0.025 (2)	−0.0009 (17)	0.008 (2)
C4M'	0.039 (6)	0.085 (6)	0.051 (6)	0.014 (6)	0.006 (5)	0.013 (6)
C5M'	0.052 (6)	0.080 (6)	0.069 (6)	0.003 (6)	0.007 (6)	0.016 (6)
C6M'	0.059 (8)	0.082 (7)	0.073 (7)	−0.007 (7)	0.014 (7)	0.026 (7)
C7M'	0.088 (14)	0.110 (15)	0.100 (14)	−0.004 (14)	−0.003 (13)	−0.003 (14)
C8M'	0.075 (13)	0.103 (15)	0.136 (15)	−0.004 (14)	0.027 (13)	0.017 (14)

### Geometric parameters (Å, º)

**Table:**

N1—C2	1.340 (4)	C10—H10	1.0000
N1—C12B	1.464 (4)	C11—C12	1.539 (5)
N1—H1	0.87 (4)	C11—H11A	0.9900
C2—O2	1.232 (4)	C11—H11B	0.9900
C2—C3	1.492 (5)	C12—C12A	1.536 (5)
C3—C3A	1.549 (4)	C12—H12F	0.9900
C3—H3A	0.9900	C12—H12G	0.9900
C3—H3B	0.9900	C12A—C12B	1.540 (4)
C3A—C4	1.571 (5)	C12A—C121	1.554 (5)
C3A—C7A	1.584 (4)	C12B—H12B	1.0000
C3A—H3C	1.0000	C121—H12C	0.9800
C4—C41	1.530 (5)	C121—H12D	0.9800
C4—C5	1.533 (5)	C121—H12E	0.9800
C4—C42	1.550 (4)	C1'—C2'	1.529 (6)
C41—H41A	0.9800	C1'—H1A'	0.9800
C41—H41B	0.9800	C1'—H1B'	0.9800
C41—H41C	0.9800	C1'—H1C'	0.9800
C42—H42A	0.9800	C2'—C3M'	1.523 (5)
C42—H42B	0.9800	C2'—C3'	1.523 (5)
C42—H42C	0.9800	C2'—H2'	1.0000
C5—O51	1.448 (4)	C3'—C4'	1.552 (6)
C5—C6	1.506 (4)	C3'—H3A'	0.9900
C5—H5	1.0000	C3'—H3B'	0.9900
O51—C51	1.348 (4)	C4'—C5'	1.492 (7)
C51—O52	1.209 (5)	C4'—H4A'	0.9900
C51—C52	1.492 (5)	C4'—H4B'	0.9900
C52—H52A	0.9800	C5'—C6'	1.519 (8)
C52—H52B	0.9800	C5'—H5A'	0.9900
C52—H52C	0.9800	C5'—H5B'	0.9900
C6—C7	1.516 (4)	C6'—C8'	1.474 (10)
C6—H6A	0.9900	C6'—C7'	1.583 (9)
C6—H6B	0.9900	C6'—H6'	1.0000
C7—C7A	1.553 (4)	C7'—H7A'	0.9800
C7—H7A	0.9900	C7'—H7B'	0.9800
C7—H7B	0.9900	C7'—H7C'	0.9800
C7A—C71	1.536 (4)	C8'—H8A'	0.9800
C7A—C7B	1.599 (5)	C8'—H8B'	0.9800
C71—H71A	0.9800	C8'—H8C'	0.9800
C71—H71B	0.9800	C3M'—C4M'	1.554 (9)
C71—H71C	0.9800	C3M'—H3C'	0.9900
C7B—O7	1.427 (4)	C3M'—H3D'	0.9900
C7B—C12B	1.578 (4)	C4M'—C5M'	1.497 (10)
C7B—C8	1.580 (4)	C4M'—H4C'	0.9900
O7—H7	0.79 (5)	C4M'—H4D'	0.9900
C8—O8	1.196 (4)	C5M'—C6M'	1.506 (10)
C8—C9	1.549 (5)	C5M'—H5C'	0.9900
C9—O9	1.202 (4)	C5M'—H6D'	0.9900
C9—C9A	1.500 (4)	C6M'—C8M'	1.474 (12)
C9A—C91	1.542 (4)	C6M'—C7M'	1.588 (11)
C9A—C10	1.548 (4)	C6M'—H6M'	1.0000
C9A—C12A	1.566 (4)	C7M'—H7D'	0.9800
C91—H91A	0.9800	C7M'—H7E'	0.9800
C91—H91B	0.9800	C7M'—H7F'	0.9800
C91—H91C	0.9800	C8M'—H8D'	0.9800
C10—C2'	1.554 (4)	C8M'—H8E'	0.9800
C10—C11	1.557 (5)	C8M'—H8F'	0.9800
			
C2—N1—C12B	125.6 (3)	H11A—C11—H11B	108.4
C2—N1—H1	121 (3)	C12A—C12—C11	104.8 (3)
C12B—N1—H1	113 (3)	C12A—C12—H12F	110.8
O2—C2—N1	120.6 (3)	C11—C12—H12F	110.8
O2—C2—C3	123.5 (3)	C12A—C12—H12G	110.8
N1—C2—C3	115.6 (3)	C11—C12—H12G	110.8
C2—C3—C3A	107.1 (3)	H12F—C12—H12G	108.9
C2—C3—H3A	110.3	C12—C12A—C12B	112.7 (3)
C3A—C3—H3A	110.3	C12—C12A—C121	108.8 (3)
C2—C3—H3B	110.3	C12B—C12A—C121	112.3 (3)
C3A—C3—H3B	110.3	C12—C12A—C9A	101.1 (2)
H3A—C3—H3B	108.6	C12B—C12A—C9A	111.2 (2)
C3—C3A—C4	110.6 (2)	C121—C12A—C9A	110.2 (3)
C3—C3A—C7A	114.4 (3)	N1—C12B—C12A	107.9 (2)
C4—C3A—C7A	118.0 (3)	N1—C12B—C7B	110.7 (2)
C3—C3A—H3C	104.0	C12A—C12B—C7B	115.7 (3)
C4—C3A—H3C	104.0	N1—C12B—H12B	107.4
C7A—C3A—H3C	104.0	C12A—C12B—H12B	107.4
C41—C4—C5	112.4 (3)	C7B—C12B—H12B	107.4
C41—C4—C42	107.6 (3)	C12A—C121—H12C	109.5
C5—C4—C42	107.7 (3)	C12A—C121—H12D	109.5
C41—C4—C3A	114.9 (3)	H12C—C121—H12D	109.5
C5—C4—C3A	106.2 (2)	C12A—C121—H12E	109.5
C42—C4—C3A	107.7 (2)	H12C—C121—H12E	109.5
C4—C41—H41A	109.5	H12D—C121—H12E	109.5
C4—C41—H41B	109.5	C2'—C1'—H1A'	109.5
H41A—C41—H41B	109.5	C2'—C1'—H1B'	109.5
C4—C41—H41C	109.5	H1A'—C1'—H1B'	109.5
H41A—C41—H41C	109.5	C2'—C1'—H1C'	109.5
H41B—C41—H41C	109.5	H1A'—C1'—H1C'	109.5
C4—C42—H42A	109.5	H1B'—C1'—H1C'	109.5
C4—C42—H42B	109.5	C3M'—C2'—C1'	110.8 (3)
H42A—C42—H42B	109.5	C3'—C2'—C1'	110.8 (3)
C4—C42—H42C	109.5	C3M'—C2'—C10	111.6 (3)
H42A—C42—H42C	109.5	C3'—C2'—C10	111.6 (3)
H42B—C42—H42C	109.5	C1'—C2'—C10	111.0 (3)
O51—C5—C6	108.5 (2)	C3'—C2'—H2'	107.8
O51—C5—C4	110.2 (3)	C1'—C2'—H2'	107.8
C6—C5—C4	112.5 (3)	C10—C2'—H2'	107.8
O51—C5—H5	108.5	C2'—C3'—C4'	111.7 (4)
C6—C5—H5	108.5	C2'—C3'—H3A'	109.3
C4—C5—H5	108.5	C4'—C3'—H3A'	109.3
C51—O51—C5	117.4 (3)	C2'—C3'—H3B'	109.3
O52—C51—O51	124.5 (3)	C4'—C3'—H3B'	109.3
O52—C51—C52	125.2 (4)	H3A'—C3'—H3B'	107.9
O51—C51—C52	110.3 (3)	C5'—C4'—C3'	112.5 (4)
C51—C52—H52A	109.5	C5'—C4'—H4A'	109.1
C51—C52—H52B	109.5	C3'—C4'—H4A'	109.1
H52A—C52—H52B	109.5	C5'—C4'—H4B'	109.1
C51—C52—H52C	109.5	C3'—C4'—H4B'	109.1
H52A—C52—H52C	109.5	H4A'—C4'—H4B'	107.8
H52B—C52—H52C	109.5	C4'—C5'—C6'	116.5 (5)
C5—C6—C7	109.4 (2)	C4'—C5'—H5A'	108.2
C5—C6—H6A	109.8	C6'—C5'—H5A'	108.2
C7—C6—H6A	109.8	C4'—C5'—H5B'	108.2
C5—C6—H6B	109.8	C6'—C5'—H5B'	108.2
C7—C6—H6B	109.8	H5A'—C5'—H5B'	107.3
H6A—C6—H6B	108.2	C8'—C6'—C5'	114.3 (6)
C6—C7—C7A	114.1 (3)	C8'—C6'—C7'	109.4 (6)
C6—C7—H7A	108.7	C5'—C6'—C7'	110.4 (5)
C7A—C7—H7A	108.7	C8'—C6'—H6'	107.5
C6—C7—H7B	108.7	C5'—C6'—H6'	107.5
C7A—C7—H7B	108.7	C7'—C6'—H6'	107.5
H7A—C7—H7B	107.6	C6'—C7'—H7A'	109.5
C71—C7A—C7	109.3 (3)	C6'—C7'—H7B'	109.5
C71—C7A—C3A	112.6 (3)	H7A'—C7'—H7B'	109.5
C7—C7A—C3A	107.4 (2)	C6'—C7'—H7C'	109.5
C71—C7A—C7B	109.7 (3)	H7A'—C7'—H7C'	109.5
C7—C7A—C7B	107.7 (3)	H7B'—C7'—H7C'	109.5
C3A—C7A—C7B	110.0 (2)	C6'—C8'—H8A'	109.5
C7A—C71—H71A	109.5	C6'—C8'—H8B'	109.5
C7A—C71—H71B	109.5	H8A'—C8'—H8B'	109.5
H71A—C71—H71B	109.5	C6'—C8'—H8C'	109.5
C7A—C71—H71C	109.5	H8A'—C8'—H8C'	109.5
H71A—C71—H71C	109.5	H8B'—C8'—H8C'	109.5
H71B—C71—H71C	109.5	C2'—C3M'—C4M'	134.8 (10)
O7—C7B—C12B	109.8 (2)	C2'—C3M'—H3C'	103.5
O7—C7B—C8	97.7 (2)	C4M'—C3M'—H3C'	103.5
C12B—C7B—C8	109.7 (2)	C2'—C3M'—H3D'	103.5
O7—C7B—C7A	112.5 (2)	C4M'—C3M'—H3D'	103.5
C12B—C7B—C7A	113.8 (3)	H3C'—C3M'—H3D'	105.3
C8—C7B—C7A	112.2 (2)	C5M'—C4M'—C3M'	104.0 (9)
C7B—O7—H7	106 (3)	C5M'—C4M'—H4C'	111.0
O8—C8—C9	115.9 (3)	C3M'—C4M'—H4C'	111.0
O8—C8—C7B	124.1 (3)	C5M'—C4M'—H4D'	111.0
C9—C8—C7B	119.9 (3)	C3M'—C4M'—H4D'	111.0
O9—C9—C9A	128.7 (3)	H4C'—C4M'—H4D'	109.0
O9—C9—C8	119.0 (3)	C4M'—C5M'—C6M'	120.1 (10)
C9A—C9—C8	112.3 (3)	C4M'—C5M'—H5C'	107.3
C9—C9A—C91	107.6 (3)	C6M'—C5M'—H5C'	107.3
C9—C9A—C10	118.8 (3)	C4M'—C5M'—H6D'	107.3
C91—C9A—C10	111.4 (3)	C6M'—C5M'—H6D'	107.3
C9—C9A—C12A	102.5 (2)	H5C'—C5M'—H6D'	106.9
C91—C9A—C12A	113.4 (3)	C8M'—C6M'—C5M'	116.1 (12)
C10—C9A—C12A	102.9 (2)	C8M'—C6M'—C7M'	108.3 (11)
C9A—C91—H91A	109.5	C5M'—C6M'—C7M'	109.0 (11)
C9A—C91—H91B	109.5	C8M'—C6M'—H6M'	107.7
H91A—C91—H91B	109.5	C5M'—C6M'—H6M'	107.7
C9A—C91—H91C	109.5	C7M'—C6M'—H6M'	107.7
H91A—C91—H91C	109.5	C6M'—C7M'—H7D'	109.5
H91B—C91—H91C	109.5	C6M'—C7M'—H7E'	109.5
C9A—C10—C2'	116.7 (3)	H7D'—C7M'—H7E'	109.5
C9A—C10—C11	102.2 (3)	C6M'—C7M'—H7F'	109.5
C2'—C10—C11	113.4 (3)	H7D'—C7M'—H7F'	109.5
C9A—C10—H10	108.0	H7E'—C7M'—H7F'	109.5
C2'—C10—H10	108.0	C6M'—C8M'—H8D'	109.5
C11—C10—H10	108.0	C6M'—C8M'—H8E'	109.5
C12—C11—C10	108.0 (3)	H8D'—C8M'—H8E'	109.5
C12—C11—H11A	110.1	C6M'—C8M'—H8F'	109.5
C10—C11—H11A	110.1	H8D'—C8M'—H8F'	109.5
C12—C11—H11B	110.1	H8E'—C8M'—H8F'	109.5
C10—C11—H11B	110.1		
			
C12B—N1—C2—O2	175.9 (3)	C8—C9—C9A—C10	176.1 (3)
C12B—N1—C2—C3	1.9 (4)	O9—C9—C9A—C12A	−115.8 (4)
O2—C2—C3—C3A	−101.2 (3)	C8—C9—C9A—C12A	63.6 (3)
N1—C2—C3—C3A	72.6 (3)	C9—C9A—C10—C2'	85.6 (4)
C2—C3—C3A—C4	131.8 (3)	C91—C9A—C10—C2'	−40.3 (4)
C2—C3—C3A—C7A	−92.1 (3)	C12A—C9A—C10—C2'	−162.1 (3)
C3—C3A—C4—C41	60.2 (3)	C9—C9A—C10—C11	−150.0 (3)
C7A—C3A—C4—C41	−74.3 (4)	C91—C9A—C10—C11	84.1 (3)
C3—C3A—C4—C5	−174.8 (2)	C12A—C9A—C10—C11	−37.7 (3)
C7A—C3A—C4—C5	50.7 (3)	C9A—C10—C11—C12	15.6 (4)
C3—C3A—C4—C42	−59.7 (3)	C2'—C10—C11—C12	142.1 (3)
C7A—C3A—C4—C42	165.8 (3)	C10—C11—C12—C12A	13.0 (4)
C41—C4—C5—O51	−52.8 (3)	C11—C12—C12A—C12B	−154.6 (3)
C42—C4—C5—O51	65.6 (3)	C11—C12—C12A—C121	80.2 (3)
C3A—C4—C5—O51	−179.3 (2)	C11—C12—C12A—C9A	−35.8 (3)
C41—C4—C5—C6	68.4 (4)	C9—C9A—C12A—C12	170.1 (3)
C42—C4—C5—C6	−173.2 (3)	C91—C9A—C12A—C12	−74.3 (3)
C3A—C4—C5—C6	−58.1 (3)	C10—C9A—C12A—C12	46.2 (3)
C6—C5—O51—C51	124.5 (3)	C9—C9A—C12A—C12B	−70.1 (3)
C4—C5—O51—C51	−112.0 (3)	C91—C9A—C12A—C12B	45.6 (3)
C5—O51—C51—O52	4.1 (5)	C10—C9A—C12A—C12B	166.1 (3)
C5—O51—C51—C52	−175.2 (3)	C9—C9A—C12A—C121	55.0 (3)
O51—C5—C6—C7	−173.2 (3)	C91—C9A—C12A—C121	170.7 (3)
C4—C5—C6—C7	64.7 (3)	C10—C9A—C12A—C121	−68.8 (3)
C5—C6—C7—C7A	−59.9 (3)	C2—N1—C12B—C12A	159.7 (3)
C6—C7—C7A—C71	−73.1 (3)	C2—N1—C12B—C7B	−72.9 (4)
C6—C7—C7A—C3A	49.3 (3)	C12—C12A—C12B—N1	−66.3 (3)
C6—C7—C7A—C7B	167.8 (2)	C121—C12A—C12B—N1	57.0 (3)
C3—C3A—C7A—C71	−59.0 (4)	C9A—C12A—C12B—N1	−179.1 (3)
C4—C3A—C7A—C71	73.8 (3)	C12—C12A—C12B—C7B	169.1 (3)
C3—C3A—C7A—C7	−179.3 (3)	C121—C12A—C12B—C7B	−67.5 (3)
C4—C3A—C7A—C7	−46.5 (3)	C9A—C12A—C12B—C7B	56.4 (3)
C3—C3A—C7A—C7B	63.8 (3)	O7—C7B—C12B—N1	−48.4 (3)
C4—C3A—C7A—C7B	−163.4 (2)	C8—C7B—C12B—N1	−154.7 (3)
C71—C7A—C7B—O7	−166.2 (3)	C7A—C7B—C12B—N1	78.7 (3)
C7—C7A—C7B—O7	−47.4 (3)	O7—C7B—C12B—C12A	74.6 (3)
C3A—C7A—C7B—O7	69.4 (3)	C8—C7B—C12B—C12A	−31.6 (4)
C71—C7A—C7B—C12B	68.1 (3)	C7A—C7B—C12B—C12A	−158.2 (2)
C7—C7A—C7B—C12B	−173.1 (2)	C9A—C10—C2'—C3M'	169.8 (4)
C3A—C7A—C7B—C12B	−56.3 (3)	C11—C10—C2'—C3M'	51.4 (5)
C71—C7A—C7B—C8	−57.2 (3)	C9A—C10—C2'—C3'	169.8 (4)
C7—C7A—C7B—C8	61.7 (3)	C11—C10—C2'—C3'	51.4 (5)
C3A—C7A—C7B—C8	178.5 (3)	C9A—C10—C2'—C1'	−66.1 (4)
O7—C7B—C8—O8	88.8 (5)	C11—C10—C2'—C1'	175.5 (3)
C12B—C7B—C8—O8	−156.9 (4)	C1'—C2'—C3'—C4'	66.8 (6)
C7A—C7B—C8—O8	−29.4 (5)	C10—C2'—C3'—C4'	−169.0 (4)
O7—C7B—C8—C9	−87.4 (3)	C2'—C3'—C4'—C5'	163.9 (5)
C12B—C7B—C8—C9	26.9 (4)	C3'—C4'—C5'—C6'	168.9 (5)
C7A—C7B—C8—C9	154.3 (3)	C4'—C5'—C6'—C8'	−177.4 (7)
O8—C8—C9—O9	−43.7 (5)	C4'—C5'—C6'—C7'	58.7 (8)
C7B—C8—C9—O9	132.8 (3)	C1'—C2'—C3M'—C4M'	33.6 (11)
O8—C8—C9—C9A	136.8 (4)	C10—C2'—C3M'—C4M'	157.8 (10)
C7B—C8—C9—C9A	−46.7 (4)	C2'—C3M'—C4M'—C5M'	82 (2)
O9—C9—C9A—C91	124.4 (4)	C3M'—C4M'—C5M'—C6M'	170 (2)
C8—C9—C9A—C91	−56.2 (3)	C4M'—C5M'—C6M'—C8M'	−27 (3)
O9—C9—C9A—C10	−3.4 (5)	C4M'—C5M'—C6M'—C7M'	−150 (3)

### Hydrogen-bond geometry (Å, º)

**Table:**

*D*—H···*A*	*D*—H	H···*A*	*D*···*A*	*D*—H···*A*
O7—H7···O2^i^	0.79 (5)	2.02 (5)	2.750 (3)	153 (5)
C3*A*—H3*C*···O2^i^	1.00	2.18	3.173 (4)	176
C121—H12*E*···O52^ii^	0.98	2.53	3.428 (4)	153
C71—H71*A*···O8^iii^	0.98	2.55	3.224 (4)	126
C91—H91*B*···O9^iv^	0.98	2.48	3.422 (5)	162

Symmetry codes: (i) −*x*+3/2, *y*+1/2, −*z*; (ii) −*x*+3/2, *y*−1/2, −*z*; (iii) −*x*+3/2, *y*−1/2, −*z*+1; (iv) *x*, *y*−1, *z*.
